# Caspase-8 but not caspase-7 influences inflammasome activation to act in control of *Brucella abortus* infection

**DOI:** 10.3389/fmicb.2022.1086925

**Published:** 2022-12-01

**Authors:** Raiany A. Santos, Daiane M. Cerqueira, Dario S. Zamboni, Sergio C. Oliveira

**Affiliations:** ^1^Departamento de Genética, Ecologia e Evolução, Programa de Pós-Graduação em Genética, Instituto de Ciências Biológicas, Universidade Federal de Minas Gerais, Belo Horizonte, Minas Gerais, Brazil; ^2^Departamento de Bioquímica e Imunologia, Instituto de Ciências Biológicas, Universidade Federal de Minas Gerais, Belo Horizonte, Minas Gerais, Brazil; ^3^Departamento de Biologia Celular e Molecular e Bioagentes Patogênicos, Faculdade de Medicina de Ribeirão Preto, Universidade de São Paulo, Ribeirão Preto, Brazil; ^4^Departamento de Imunologia, Instituto de Ciências Biomédicas, Universidade de São Paulo, São Paulo, Brazil

**Keywords:** caspase-8, caspase-7, *Brucella abortus*, inflammasome, pyroptosis, innate immunity

## Abstract

Programmed cell death (PCD) is an important mechanism of innate immunity against bacterial pathogens. The innate immune PCD pathway involves the molecules caspase-7 and caspase-8, among others. *Brucella abortus* is a gram-negative bacterium that causes a zoonotic disease termed brucellosis. The innate immune response against this pathogen involves activation of inflammasome components and induction of pyroptosis. However, no studies so far have revealed the role of caspase-7 or caspase-8 during this bacterial infection. Herein, we demonstrate that caspase-7 is dispensable for caspase-1 processing, IL-1β secretion and cell death in macrophages. Additionally, caspase-7 deficient animals control *B. abortus* infection as well as the wild type mice. Furthermore, we addressed the role of caspase-8 in inflammasome activation and pyroptosis during this bacterial infection. Macrophages deficient in caspase-8 secreted reduced amounts of IL-1β that parallels with diminished caspase-1 activity when compared to wild type cells. Additionally, caspase-8 KO macrophages showed reduced LDH release when compared to wild type, suggesting that caspase-8 may play an important role in pyroptosis in response to *B. abortus*. Finally, caspase-8 KO animals were more susceptible to *Brucella* infection when compared to wild type mice. Overall, this study contributes to a better understanding of the involvement of caspase-7 and caspase-8 in innate immunity against *B. abortus* infection.

## Introduction

Brucellosis is a zoonosis caused by bacteria of the genus *Brucella* of worldwide distribution. Great advance has been made in the control of brucellosis in recent years. However, in many regions of the world, *Brucella* infection in domestic animals still persists, leading to frequent transmission to the human population. In regions such as the Mediterranean countries of Europe, like Portugal, Italy and Greece, brucellosis is still considered an important human disease and it is often neglected (Corbel, 1997).

Innate immunity is an important arm of the immune system involved in the control of *Brucella* infection. In previous studies performed by our research group, we demonstrated that receptors and adaptor molecules such as TLR9, AIM2, MyD88, and STING are important components in the protective response against *Brucella* infection (Macedo et al., 2008; Gomes et al., 2016; Costa Franco et al., 2018, 2019). In addition, we and others have shown that pyroptosis triggered upon activation of inflammasomes, is also an important mechanism in restricting *in vivo* infection against *Brucella abortus* (Cerqueira et al., 2018; Lacey et al., 2018). In recent study, we showed that caspase-11/GSDMD-dependent pyroptosis process triggered by *B. abortus* contributed to the restriction of infection *in vivo* by assisting in the recruitment and activation of immune cells such as neutrophils, macrophages, and dendritic cells (Cerqueira et al., 2018).

Programmed cell death (PCD) is a intricate circuit that involves the cross-talk among different caspases, and their substrates. Caspase-3 and caspase-7 are considered executioner molecules triggering host cell apoptosis (Kim et al., 2005). Recent study demonstrated that *Brucella* inhibited the PCD in early stage of infection to allow bacterial replication in host cells and promoted apoptosis in the later stage during infection in macrophages (Zhang et al., 2022). In the literature, there are some controversies in the role of caspase-7 during bacterial infections. Caspase-7 has been implicated in resistance to *L. pneumophila* through the NLRC4 inflammasome (Akhter et al., 2009). In contrast, Gonçalves et al. (Goncalves et al., 2019) demonstrated that mice with a single deletion in caspase-7 are not fully susceptible to *L. pneumophila*. The *Casp7^−/−^* did not phenocopy the susceptibility to *L. pneumophila* infection as observed in *Nlrc4^−/−^* animals.

Caspase-8, early on classified as an apoptotic caspase, has lately been shown to have a role in several inflammatory processes. Caspase-8 is involved in the inflammasome pathway and can be activated by NLRP3, AIM2 and NLRC4 in macrophages (Man et al., 2013; Sagulenko et al., 2013) and NLRP3 inflammasome in dendritic cells (Antonopoulos et al., 2015). Caspase-8 can act by controlling NF-kB signaling, influencing the positive regulation of components of the inflammasome, such as the NLRP3 and pro-IL-1β (Weng et al., 2014). It can also activate the inflammasome pathway in response to *C. albicans* β-glucans (Ganesan et al., 2014). Interestingly, upon TLR or death receptor activation, active caspase-8 can cleave the IL-1β precursor into its bioactive fragment at the same site as caspase-1 (Shenderov et al., 2014), and can directly cleave GSDMD into its N-terminal fragment, triggering pyroptosis during *Yersinia* infection (Sarhan et al., 2018). In addition, once the inflammasome is activated, but pyroptosis is impaired, caspase-8 can act leading to a cell death program. This has been demonstrated in studies with intracellular bacteria such as *L. pneumophila* and *S. Typhimurium* in the absence of caspase-1 or GSDMD (Mascarenhas et al., 2017; Lee et al., 2018).

To the best of our knowledge, the role of caspase-7 and caspase-8 in *Brucella* infection has not been addressed so far. Therefore, in order to expand the understanding of the mechanisms involved in the innate immune response and inflammatory cell death, we investigated the participation of the caspase-7 and caspase-8 molecules during *B. abortus* infection.

## Materials and methods

### Mice

Wild-type C57BL/6 (WT) mice were purchased from the Federal University of Minas Gerais (UFMG) and*, Casp7^−/−^, Casp7/1/11^−/−^, Casp7/Gsdmd^−/−^, Gsdmd^−/−^*, *Casp8*^+/+^*/RIPK3^−/−^*, and *Casp8/RIPK3^−/−^* were kindly provided by Dr. Prof. Dario Simões Zamboni, Department of Cell and Molecular Biology and Pathogenic Bioagents, Ribeirão Preto Medical School, University of Sao Paulo, Brazil. Genetically deficient and control mice were maintained at our facilities and used at 6–8 weeks of age. Mice were housed in filter top cages and provided with sterile water and food *ad libitum*. Groups of 5–7 animals were used to perform all experiments. The procedures for animal experimentation were approved by the Ethics Committee for the Use of Animals of the Federal University of Minas Gerais-CEUA/UFMG under protocol number 69/2020.

### Bacteria and culture conditions

*Brucella abortus* virulent strain 2,308 was used in this study. To prepare the inoculum, the bacteria were grown in BB (*Brucella Broth*) medium (BD Biosciences, United States) for 24 h at 37°C under 180 rpm shaking, washed in PBS for 10 min, 5,000 rpm at 4°C, and resuspended in sterile PBS. The OD of the culture was measured at 600 nm in a spectrophotometer to determine the bacterial number in the solution.

### Mice infection with *Brucella abortus*

Five to seven mice from each group were infected intraperitoneally (i.p.) with 1 × 10^6^ *B. abortus* in 100 μl of PBS and the animals sacrificed at 14 days post-infection. The spleens were harvested and macerated in 10 ml saline (NaCl 0.9%), serially diluted, and plated in duplicated on *Brucella Broth* agar. Plates were incubated for 3 days at 37°C and the CFU number was determined.

### Bone marrow-derived macrophages

BMDMs were differentiated *in vitro* from bone marrow cells extracted from mouse femurs. Cultures were differentiated for 7 days in an incubator at 37°C, 5% CO2 in DMEM medium supplemented with 1% HEPES, 20% fetal bovine serum (FBS), 30% L929 cell-conditioned medium (LCCM) source of M-CSF (important for differentiation of progenitor cells into macrophages), 100 U/ml penicillin and 100 μg/ml streptomycin (Thermo Fischer Scientific). After differentiation, macrophages were collected by washing the monolayers with ice-cold PBS, distributed on culture plates, and cultured in DMEM medium containing 1% SFB and 1% HEPES or 10% SFB and 1% HEPES and they were ready for use.

### Lactate dehydrogenase release assay

For the lactate dehydrogenase (LDH) release assay, BMDMs were plated at 5 × 10^5^ cells/well in 24-well plates and infected with *B. abortus* (MOI 100) for 8 h. RPMI 1640 medium without phenol red, with 1% glutamine, 1% FBS was used. Supernatants were collected, and LDH was quantified using the Cytotox96 LDH kit (Promega, Madison, WI) according to the manufacturer’s instructions. During infection, bacteria were opsonized with a polyclonal mouse antibody (anti-*B.abortus*, dilution 1:1,000) to ensure more efficient bacterial phagocytosis. This polyclonal antibody was generated by injecting 1 × 10^6^ heat-killed bacteria/mouse. The animals were injected three times during a 15-day interval, and after this period, serum from each mouse was tested for the presence of the specific antibody and stored at −80°C.

### Cytokine measurement

For cytokine determination, BMDMs were plated at a concentration of 5 × 10^5^ cells/well in 24-well plates and the cells were infected with *B. abortus* at an MOI of 100 for 17 h. Supernatants were collected and cytokines were measured with the mouse IL-1β, ELISA kit (R&D systems, Minneapolis, MN) according to the manufacturer’s instructions.

### Western blot analysis

BMDMs were cultured at 5 × 10^5^ cells/well in 24-well plates. The cells were infected with *B. abortus* for 17 h as described above. After 17 h of infection, culture supernatants were harvested and cells were lysed with M-PER Mammalian Protein Extraction Reagent (Thermo Fisher Scientific) supplemented with 1:100 protease inhibitor mixture (Sigma-Aldrich). Cell lysates and supernatants were subjected to SDS-PAGE analysis as already described in previous studies by our research group (Cerqueira et al., 2018). The primary Abs used included a mouse monoclonal against the p20 subunit of caspase-1 (Adipogen, San Diego, CA, United States) at a dilution of 1:1,000. Loading control was performed using anti-β-actin mAb (Cell Signaling Technology, Danvers, MA) at a dilution of 1:1,000.

### Statistical analysis

Statistical analysis was performed using Prism 5.0 software (GraphPad Software, San Diego, CA). The unpaired Student *t*-test was used to compare two groups. One-way ANOVA followed by multiple comparisons according to Tukey procedure was used to compare three or more groups. Unless otherwise stated, data are expressed as the mean ± SD. Differences were considered statistically significant at a *p*-value <0.05.

## Results

### Il-1β secretion in response to *Brucella abortus* occurs in a caspase-7-independent manner

**Figure 1:**
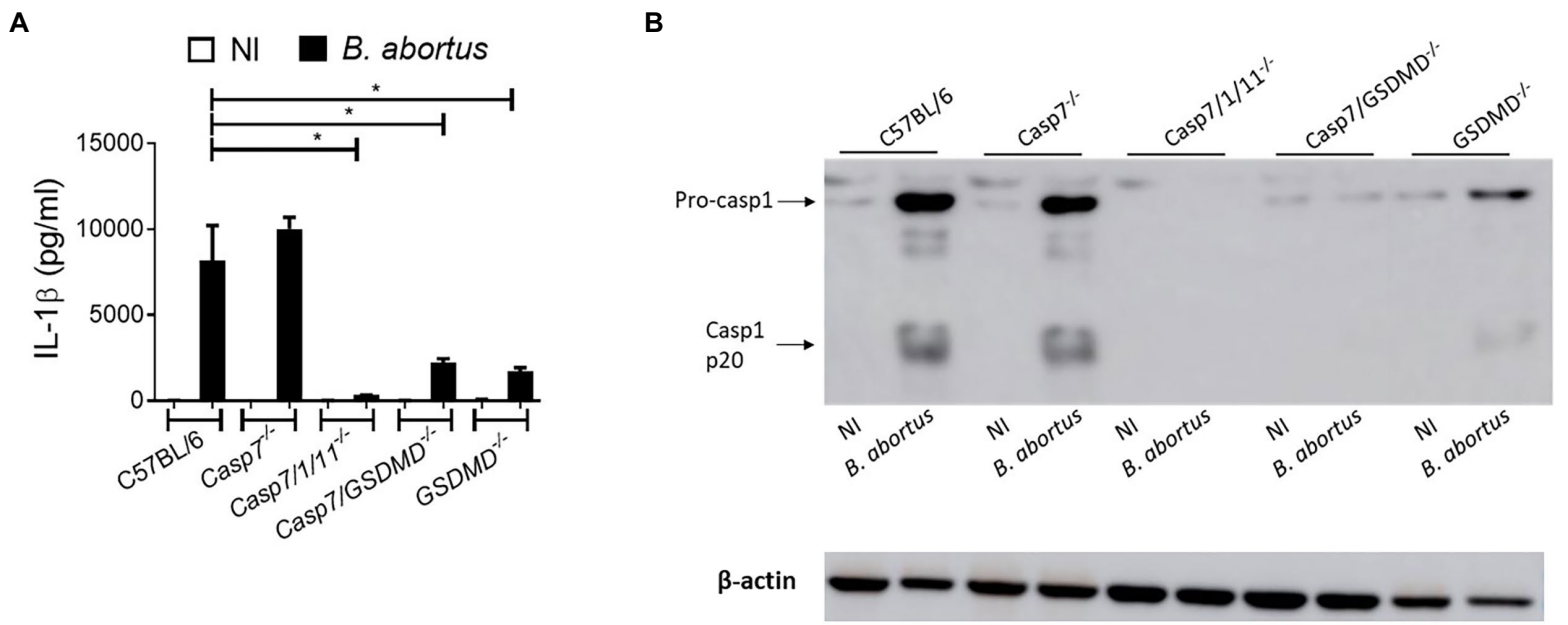
IL-1β production in response to *Brucella abortus* occurs in a caspase-7-independent manner. BMDMs were infected with *B. abortus* MOI: 100 for 17 h. **(A)** IL-1β measurement in the supernatant by ELISA. **(B)** Immunoblot analysis of caspase-1 processing. Data show the mean ± standard deviation of triplicates. The data are representative of three independent experiments. One-way ANOVA, *p < 0.05 compared to C57BL/6.

The classical executioner caspases (caspase-7 and -3) are activated to initiate the process that culminate in the classical cell death signals (Nagata, 2018). Previous studies demonstrate that caspase-7 activation requires caspase-1 processing under inflammatory conditions (Lamkanfi and Kanneganti, 2010). To investigate whether caspase-7 participates in caspase-1 cleavage and IL-1β secretion during *Brucella abortus* infection, we infected BMDMs of C57BL/6 (WT), *Casp7^−/−^*, *Casp7/1/11^−/−^*, *Casp7/Gsdmd^−/−^*, and *Gsdmd^−/−^* with *Brucella.* After 17 h of infection, we evaluated IL-1β secretion in the supernatant of the cells (Figure 1A) and the lysate was properly prepared for the assay of caspase-1 processing by Western blot analysis (Figure 1B). In all assays, C57BL/6 and *Gsdmd^−/−^* animals were used as controls, since the importance of gasdermin-D (GSDMD) for the control of *B.abortus* infection had been demonstrated previously by our research group (Cerqueira et al., 2018). We observed that BMDMs from *Casp7^−/−^* mice secreted similar amounts of IL-1β as WT animals. In macrophages from *Casp7/1/11^−/−^*, *Casp7/Gsdmd^−/−^*, and *Gsdmd^−/−^* animals the amount of IL-1β secreted was dramatically reduced compared to C57BL/6. Caspase-1 cleavage was observed only in the C57BL/6 and *Casp7^−/−^* strains, corroborating with the IL-1β cytokine secretion profile. Collectively, these data suggest that caspase-7 has no significant impact in IL-1β secretion and caspase-1 cleavage in response to *B. abortus* infection.

### Caspase-7 does not participate in macrophage pyroptosis during *Brucella abortus* infection

**Figure 2:**
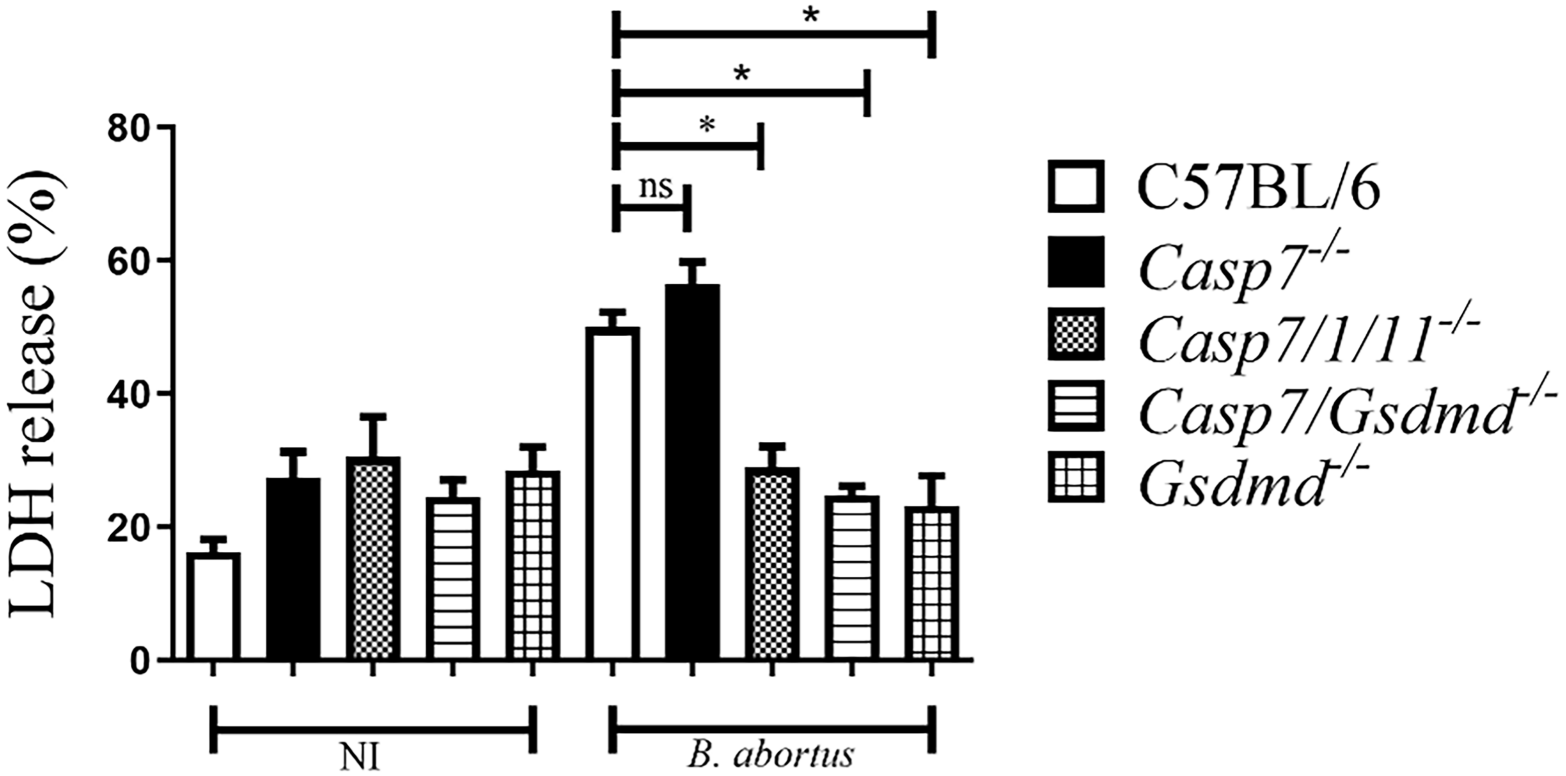
Caspase-7 does not participate in macrophage pyroptosis during *B. abortus* infection. BMDMs were infected with *Brucella abortus* MOI: 100 for 8 h and LDH quantification was performed in the cell supernatant. Values represent the percentage of LDH released compared to control cells lysed with Triton X-100. The data show the mean ± standard deviation representative of three independent experiments. One-way ANOVA, *p < 0.05 compared to C57BL/6.

Next, we addressed the role of caspase-7 in *B. abortus* induced cell death by quantifying LDH release in cell culture supernatants (Figure 2). BMDMs from C57BL/6, *Casp7^−/−^*, *Casp7/1/11^−/−^*, *Casp7/Gsdmd^−/−^*, and *Gsdmd^−/−^* mice were infected with *B.abortus* and after 8 h of infection, LDH was quantified in the supernatant. *B. abortus* infection triggered higher LDH release in BMDMs of C57BL6 and *Casp7^−/−^* strains when compared to *Casp7/1/11^−/−^*, *Casp7/Gsdmd^−/−^*, and *Gsdmd^−/−^* cells. This finding suggests that caspase-7 does not have a role in the induction of programmed cell death in response to *B. abortus* infection.

### Caspase-7 plays no role in *Brucella abortus* infection *in vivo*

**Figure 3:**
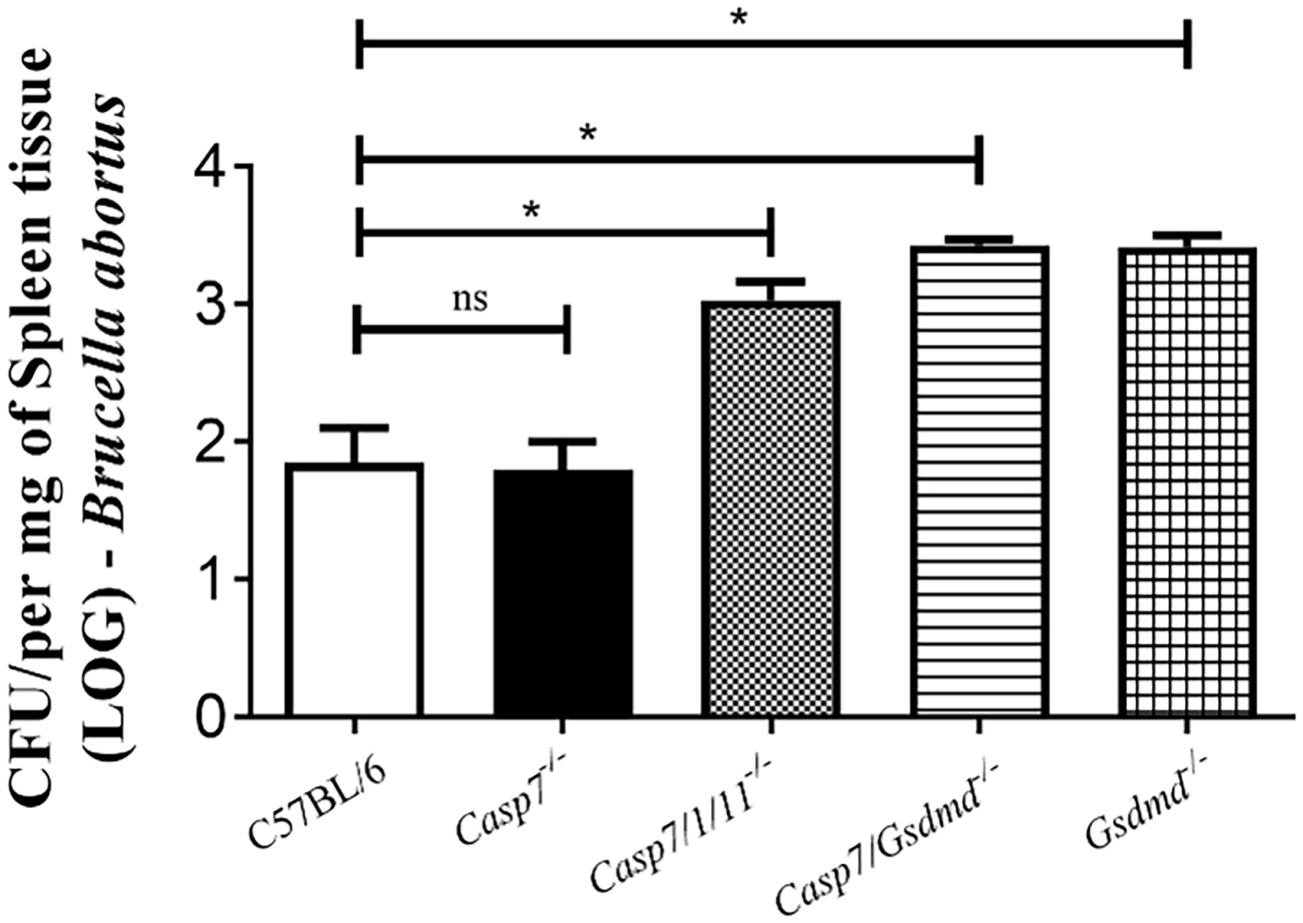
The role of caspase-7 in controlling *Brucella abortus* infection *in vivo.* Mice were infected intraperitoneally with 1 × 10^6^ CFU of *B. abortus* and sacrificed after 2 weeks of infection, and spleen homogenates were seeded onto plates containing BB agar medium for CFU determination. Data shown are the mean ± standard deviation of five mice/group. The data are representative of three independent experiments. One-way ANOVA, *p < 0.05, compared to wild-type mice.

To determine whether the absence of caspase-7 influences the control of *Brucella* infection *in vivo*, we infected C57BL/6, *Casp7^−/−^*, *Casp7/1/11^−/−^*, *Casp7/Gsdmd^−/−^*, and *Gsdmd^−/−^* mouse strains intraperitoneally and after 2 weeks the animals were sacrificed and the spleens were removed for quantification of the number of bacterial CFU. As shown in Figure 3, the bacterial burden measured in *Casp7^−/−^* animals showed no difference compared to the C57BL/6 controls. Higher bacterial numbers were observed in *Casp7/1/11^−/−^*, *Casp7/Gsdmd^−/−^*, and *Gsdmd^−/−^* mice. This result demonstrates that this susceptibility profile to infection occurred not because the lack of caspase-7, but rather, because of the deletion of *Casp1/11* or *Gsdmd*.

### Caspase-8 participates in caspase-1 cleavage and IL-1β secretion in response to *Brucella abortus*

**Figure 4:**
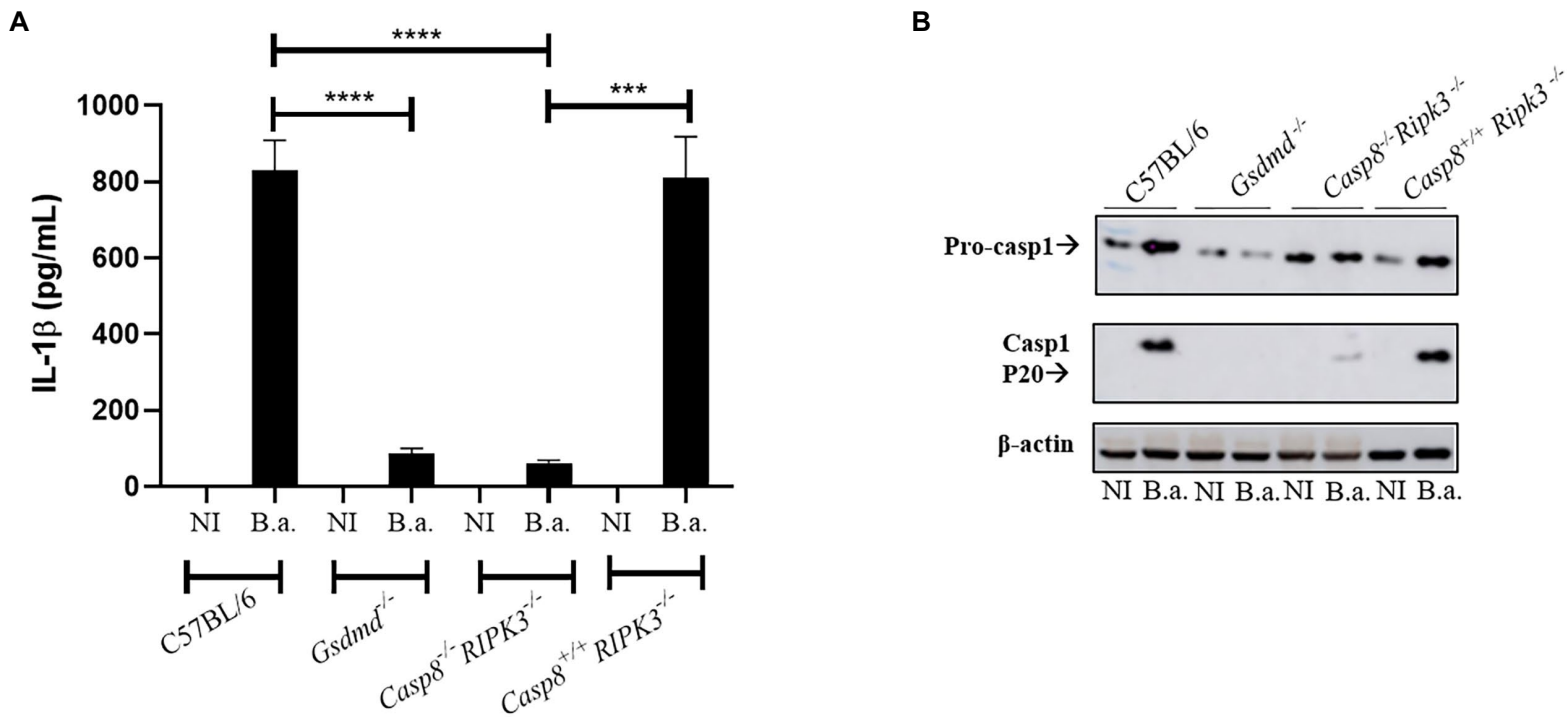
Caspase-8 participates in caspase-1 cleavage and IL-1β secretion in response to *B. abortus*. BMDMs obtained from C57BL/6, *Gsdmd^−/−^, Casp8/RIPK3*^−/−^ and *Casp8^+/+^/RIPK3^−/−^* mice were uninfected (NI) or infected with *B. abortus* S2308 with MOI 100 for 17 h. The supernatant was collected and subjected to ELISA assay to estimate the concentration of IL-1β **(A)**. The supernatant was labeled with anti-caspase-1 p20 monoclonal antibody **(B)**. Data show the mean ± the standard deviation of triplicates. The data are representative of three independent experiments. Student *t*-test, ****p < 0.0001 compared to C57BL/6 or *Casp8^+/+^/RIPK3^−/−^*. ***p <0.001.

Further, we addressed the role of caspase-8 in regulating the inflammatory response during *B.abortus* infection. Deletion of caspase-8 results in RIPK3-dependent embryonic lethality. To rescue this viability, additional deletion of the RIPK3 kinase *via* the CRISPR/Cas9 technique was required (Wang et al., 2013). Therefore, the animals used in this study deficient for caspase-8 possess the additional deletion of RIPK3. We infected BMDMs of C57BL/6, *Gsdmd^−/−^*, *Casp8/RIPK3^−/−^*, and *Casp8^+/+^/RIPK3^−/−^* mouse strains with *B. abortus*, and after 17 h of infection, we evaluated the secretion of IL-1β in the supernatant of the cells. Additionally, cell lysates were properly prepared for caspase-1 processing by Western blot analysis. BMDMs of *Casp8/RIPK3^−/−^* secreted reduced amounts of IL-1β compared to the WT and *Casp8^+/+^/RIPK3^−/−^* controls, similar to the profile observed in *Gsdmd^−/−^* macrophages (Figure 4A). Further, the immunoblot data corroborate with the IL-1β secretion profile, where caspase-1 cleavage was detected only in the C57BL/6 and *Casp8^+/+^/RIPK3^−/−^* cells (Figure 4B). These data suggest an important role of caspase-8 in caspase-1 cleavage and consequent IL-1β secretion during *B. abortus* infection.

### Lack of caspase-8 interferes with cell death induced by *Brucella abortus* infection

**Figure 5:**
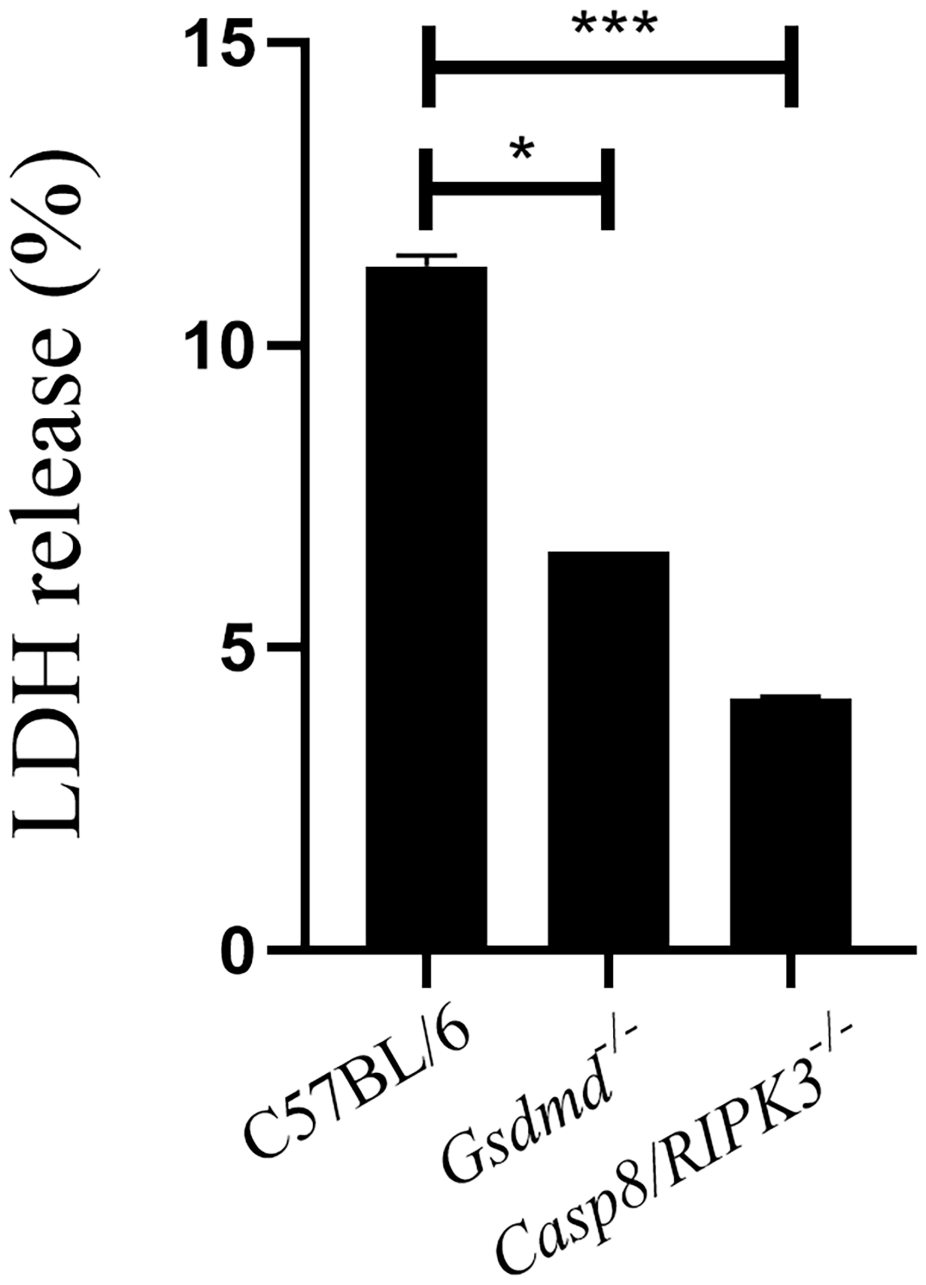
Caspase-8 is important to induce cell death during *B.abortus* infection. BMDMs of C57BL/6, *Gsdmd^−/−^* and *Casp8/RIPK3^−/−^* were infected with *B. abortus* with MOI 100 for 8hs and LDH quantification was performed in the cell supernatant. Values represent the percentage of LDH released compared to control cells lysed with Triton X-100. Data show the mean ± the representative standard deviation of triplicates. The data are representative of three independent experiments. Student *t*-test, *p < 0.05, ***p < 0.001 compared to C57BL/6.

Our results demonstrate that caspase-8 influences inflammasome activation induced by *Brucella*, since caspase-1 activation and IL-1β secretion occurs in a caspase-8-dependent manner. Thus, we sought to investigate whether caspase-8 is involved in pyroptosis induced by *B.abortus*. Macrophages from C57BL/6, *Gsdmd^−/−^*, and *Casp8/RIPK3^−/−^* strains were infected with *B.abortus* for 8 h. After this period, we performed the quantification of LDH release in cell culture supernatants (Figure 5). LDH release was greatly reduced in cells from animals deficient for caspase-8 and GSDMD, suggesting a potential role of caspase-8 in the induction of cell death in response to *B.abortus*.

### Absence of caspase-8 enhances susceptibility to *Brucella abortus* infection *in vivo*

**Figure 6:**
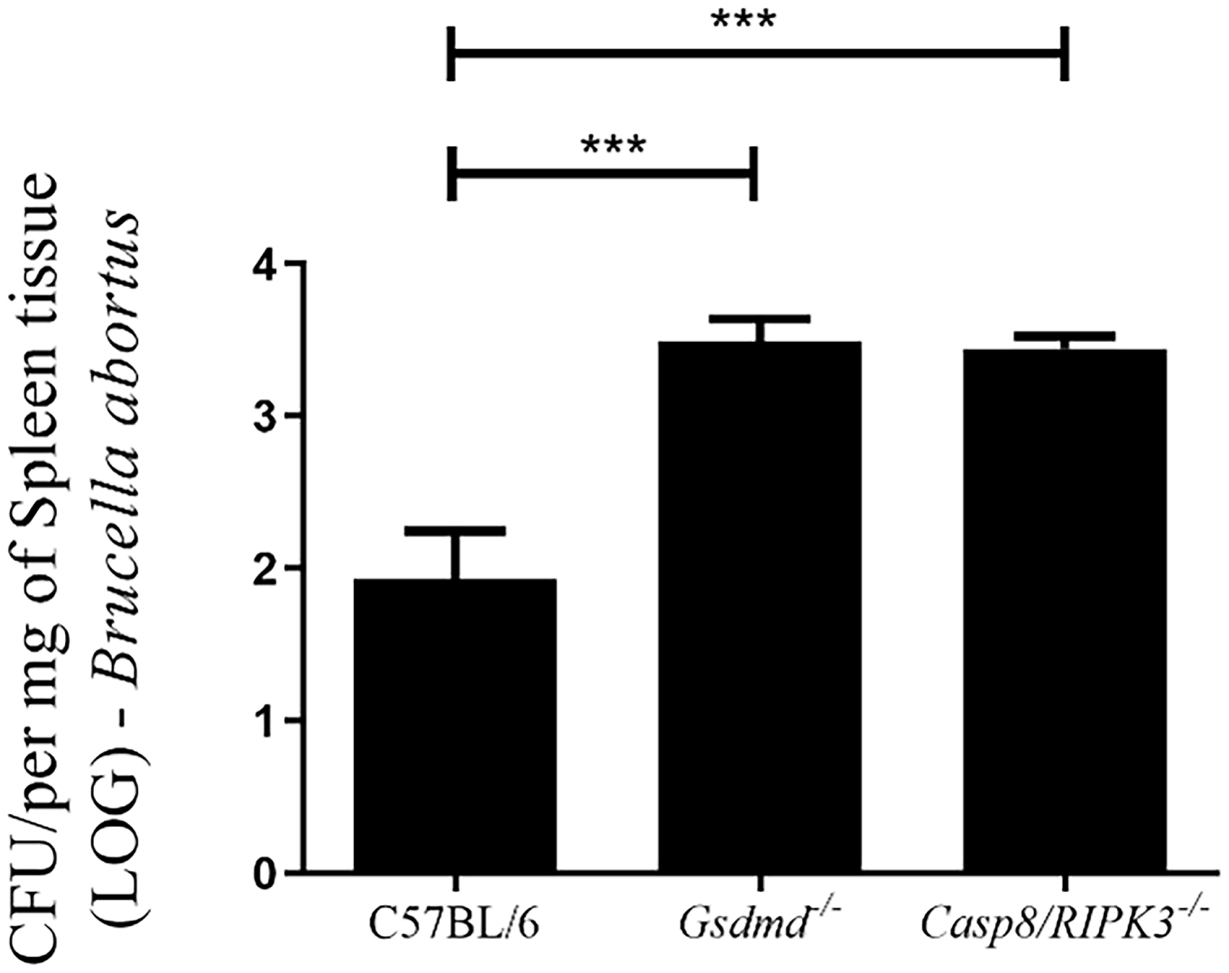
Caspase-8 influences the resistance to *Brucella* infection *in vivo*. C57BL/6, *Gsdmd^−/−^* and *Casp8/RIPK3^−/−^* m ice were infected intraperitoneally with 1×10^6^ CFU of *B. abortus*. Animals were sacrificed 2 weeks after infection and diluted spleen homogenates were plated on agar plates containing BB medium for CFU determination. Data shown are the mean ± standard deviation of five mice/group. The graph is representative of three independent experiments. One-way ANOVA, ***p < 0.001 compared to C57BL/6.

Since BMDMs deficient in caspase-8 showed reduced caspase-1 activation and secretion of IL-1β levels, we finally evaluated whether caspase-8 also played a role in restricting *Brucella* infection in mice. First, C57BL/6, *Gsdmd^−/−^*, and *Casp8/RIPK3^−/−^* mice were infected intraperitoneally with *B. abortus*. After 2 weeks of infection, bacterial colony forming units (CFU) were determined from spleen homogenates. The recovery of bacteria in the spleen of *Casp8/RIPK3^−/−^* animals was higher than the WT control group, in a manner very similar to that observed in *Gsdmd^−/−^* animals (Figure 6). Collectively, these data suggest that caspase-8 is involved in an inflammatory response and in the control of *B. abortus in vivo*.

## Discussion

Programmed cell death (PCD) can be activated in response to different stimuli (Nagata, 2018). Apoptosis, necroptosis and pyroptosis are three types of cell death that have major involvement in immune response and disease control (Schwarzer et al., 2020). Apoptosis helps in the destruction and removal of infected cells during bacterial infections (Speir et al., 2016). In the case of *Brucella*, some studies have already established that a virulent strain inhibits cell death in macrophages to allow bacterial replication (Chen et al., 2011). In contrast, in dendritic cells, astrocytes and T lymphocytes, the smooth strain induced apoptotic cell death (Garcia Samartino et al., 2010; Velasquez et al., 2012). Caspase-7, like caspase-3, is an executing caspase, both of which are activated by caspases-8 and -9 during death receptor-induced apoptosis, under certain conditions (Lamkanfi et al., 2002). Recently, some studies show that caspase-7 has a distinct role from caspase-3 during activation of apoptosis and also has a role in the inflammatory response against bacterial pathogens (Slee et al., 2001). Studies using *Salmonella typhymurium* infection of macrophages or cells stimulated with LPS and ATP, showed that caspase-7 activation was caspase-1-dependent. Additionally, Akhter et al. have observed in the absence of caspase-7 impaired ability of macrophages to restrict intracellular replication of *Legionella pneumophila* (Akhter et al., 2009). In contrast, Gonçalves et al. (Goncalves et al., 2019) demonstrated that lack of caspase-7 is not required to control *L. pneumophila* replication *in vitro* and *in vivo*. In another study, using an *in vivo* septic shock model, caspase-7-deficient mice were shown to be resistant to lethality induced by intraperitoneal injections of LPS (Lamkanfi et al., 2009). However, in our study caspase-7 does not appear to participate in the control of *B.abortus* infection. Inflammasome activation with cleavage of caspase-1 and secretion of IL-1β is not affected in the absence of caspase-7, as well as induction of cell death and *in vivo* susceptibility to infection. We have previously shown the participation of caspase-1, caspase-11 and GSDMD in controlling *B. abortus* (Cerqueira et al., 2018). Therefore, susceptibility to *Brucella* infection observed in *Casp7/1/11^−/−^* and *Casp7/Gsdmd^−/−^* animals is not due to lack of caspase-7 but rather the absence of caspase-1/11 and GSDMD.

Necroptosis is a programmed cell death pathway, with inflammatory features, that involves the kinases RIPK1 and RIPK3 and the pore-forming pseudokinase MLKL. When RIPK3 is phosphorylated, oligomerization of MLKL is initiated and subsequently inserts into the plasma membrane of the cell, leading to pore formation and cell rupture (Tummers and Green, 2017). Caspase-8 is involved in apoptosis and pyroptosis mechanisms of cell death, and when caspase-8 is inhibited RIPK1 interacts with RIPK3 leading to necroptosis (Pandian and Kanneganti, 2022). Pyroptosis is another type of inflammatory programmed cell death triggered by inflammasome activation, and for a long time, it was considered to be caspase-1-mediated in response to bacterial challenge. However, when caspase-11 was shown to detect intracellular LPS and also serve as a trigger to pyroptosis, the role of pyroptosis expanded widely (Kayagaki et al., 2011). Herein, we observed that in WT infected cells LDH release occurs, corroborating with data from our previous study where we showed that *B. abortus* infection triggers pyroptosis, and this phenomenon is GSDMD-dependent (Cerqueira et al., 2018). Additionally, in this study, we demonstrated that cell death induced by *Brucella* was also shown to be caspase-8-dependent. Caspase-8 contributes to activation of canonical and noncanonical inflammasomes. During *Salmonella* infection, caspase-8 can be recruited to the NLRC4 inflammasome regulating IL-1β secretion, but not playing a role in cell death (Man et al., 2013). In contrast, in *Yersinia* infection model, like we observed in this study, caspase-8 activates GSDMD to induce cell death (Sarhan et al., 2018). Furthermore, we observed here that mice deficient for caspase-8 and GSDMD are more susceptible to *Brucella* infection *in vivo* compared to wild type animals, suggesting that pyroptosis triggered during *B. abortus* infection is an important mechanism to control infection.

Several studies have already identified cellular functions for the GSDMD-mediated pore, such as secretion of molecules such as IL-1β and IL-1α and eicosanoids, which are important for recruiting neutrophils to the site of infection and promoting phagocytosis of infected cells and contributing to infection restriction (Jorgensen et al., 2016). Herein, reduced IL-1β secretion and pyroptosis observed in *Casp8/RIPK3^−/−^* mice are possible mechanisms that may contribute to increased susceptibility to infection. Although we did not investigate cell recruitment in this study, we hypothesize that innate cells recruitment to the site of infection may be impaired by the absence of pyroptosis in caspase-8 deficient animals, which could in part explain the increased bacterial load observed in these animals. Recently, caspase-8 was involved in *Aspergillus fumigatus* keratitis being critical in the recruitment of inflammatory cells and the clearance of the fungus (Wang et al., 2022). In summary, we suggest that caspase-8 plays an important role in cell death induced during *B.abortus* infection, contributing to inflammation and infection control in mice.

## Data availability statement

The raw data supporting the conclusions of this article will be made available by the authors, without undue reservation.

## Ethics statement

The animal study was reviewed and approved by the procedures for animal experimentation were approved by the Ethics Committee for the Use of Animals of the Federal University of Minas Gerais-CEUA/UFMG under protocol number 69/2020.

## Author contributions

RS performed all the experiments and wrote the manuscript. DC participated in the design of this study, provided assistance with data acquisition, data analysis, and statistical analysis. DZ participated in the design of this study and provided reagents to perform the experiments. SO participated in the design of this study, provided assistance with data acquisition and wrote and reviewed the manuscript. All authors contributed to the article and approved the submitted version.

## Funding

This work was supported by grants from the Conselho Nacional de Desenvolvimento Científico e Tecnológico to SO (CNPq, www.cnpq.br; grant# 303044/2020-9), Fundação de Amparo a Pesquisa do Estado de Minas Gerais to SO (FAPEMIG, www.fapemig.br; grants# APQ #01945/17 and Rede Mineira de Imunobiológicos #00140-16), National Institutes of Health to SO (NIH, www.nih.gov; grant# R01 AI116453).

## Conflict of interest

The authors declare that the research was conducted in the absence of any commercial or financial relationships that could be construed as a potential conflict of interest.

## Publisher’s note

All claims expressed in this article are solely those of the authors and do not necessarily represent those of their affiliated organizations, or those of the publisher, the editors and the reviewers. Any product that may be evaluated in this article, or claim that may be made by its manufacturer, is not guaranteed or endorsed by the publisher.
